# Efficacy and safety of ultra-low dose 0.005% estriol vaginal gel for the treatment of vulvovaginal atrophy in postmenopausal women with early breast cancer treated with nonsteroidal aromatase inhibitors: a phase II, randomized, double-blind, placebo-controlled trial

**DOI:** 10.1097/GME.0000000000001497

**Published:** 2020-02-10

**Authors:** Angelica Lindén Hirschberg, Pedro Sánchez-Rovira, Jesús Presa-Lorite, Miriam Campos-Delgado, Miguel Gil-Gil, Elisabet Lidbrink, Javier Suárez-Almarza, Concepción Nieto-Magro

**Affiliations:** 1 Department of Women's and Children's Health, Karolinska Institute and Department of Gynecology and Reproductive Medicine, Karolinska University Hospital, Stockholm, Sweden; 2 Department of Medical Oncology, Hospital Universitario de Jaén, Jaén, Spain. GEICAM Spanish Breast Cancer Group; 3 Department of Obstetrics and Gynecology, Hospital Universitario de Jaén, Jaén, Spain; 4 Department of Obstetrics and Gynecology, Hospital Universitari de Bellvitge, L’Hospitalet de Llobregat, Barcelona, Spain; 5 Breast Cancer Unit, Institut Català d’Oncologia, IDIBELL, L’Hospitalet de Llobregat, Barcelona, Spain. GEICAM Spanish Breast Cancer Group; 6 Patient area Breast cancer, Endocrine tumors and Sarcoma, Department of Medical Oncology, Karolinska University Hospital, Stockholm, Sweden; 7 Medical Department, Italfarmaco, S.A., Madrid, Spain.

**Keywords:** Aromatase inhibitors, Breast cancer, Estriol, Vaginal atrophy

## Abstract

**Objective:**

To assess the efficacy and safety of ultra-low dose 0.005% estriol vaginal gel in women with breast cancer receiving nonsteroidal aromatase inhibitors (NSAIs) and experiencing treatment-related vulvovaginal symptoms and signs.

**Methods:**

Women with hormone receptor-positive early breast cancer receiving NSAIs were randomized to either estriol vaginal gel or placebo for 12 weeks. Vaginal maturation, vaginal pH, and total and individual scores of symptoms and signs of vulvovaginal atrophy were assessed at baseline and at weeks 3 and 12; sexual functioning was also evaluated using the Female Sexual Functioning Index (FSFI) questionnaire, as well as circulating estrogens, follicle-stimulating hormone (FSH) and luteinizing hormone (LH).

**Results:**

Sixty-one women with a mean age of 59 years were included: 50 received 0.005% estriol vaginal gel and 11 received placebo. Active treatment significantly improved maturation value and pH, vaginal dryness and global scores of symptoms and signs. Active treatment also increased the total FSFI score and all the FSFI domains, with the exception of pain. Small oscillations were observed in FSH and LH, which remained within the postmenopausal range. Estriol levels increased initially and normalized by week 12, and estradiol and estrone remained mostly undetectable throughout the study.

**Conclusions:**

Ultra-low dose 0.005% estriol vaginal gel showed efficacy in improving the symptoms and signs of vulvovaginal atrophy. These results, together with minimal oscillations in hormonal levels throughout the treatment, support the use of ultra-low dose 0.005% estriol vaginal gel as a treatment option for vulvovaginal atrophy in women with breast cancer receiving NSAIs with an indication for treatment with vaginal estrogens.

## Abstract 2

Supplemental Digital Content is available in the text

## Video abstract

***Video Summary*:**

Breast cancer survivors (BCSs) who initiate adjuvant therapy with nonsteroidal aromatase inhibitors (NSAIs) experience various genitourinary signs and symptoms associated with menopausal stage. This clinical condition, currently encompassed within the term “genitourinary syndrome of menopause (GSM),” are mainly attributed to vulvovaginal atrophy and include genital (ie dryness, burning, and irritation), sexual (ie, lack of lubrication, discomfort, and pain), and urinary problems (ie, urgency, dysuria, and recurrent urinary tract infections).^1,2^ Intense estrogen deprivation associated with NSAI therapy increases the severity of GSM symptoms, which negatively impact patients’ quality of life and may consequently compromise their compliance with adjuvant endocrine therapy.^2-4^

Symptoms associated with vulvovaginal atrophy appear in 50% to 75% of BCSs, the most common being vaginal dryness and dyspareunia.^5-7^ To alleviate these and other vulvovaginal symptoms such as itching and burning, vaginal moisturizers and lubricants are recommended as first-line therapy.^2,4^ However, these products only provide temporary symptomatic relief, and do not restore the normal function of vaginal tissue.^2,4,8^ Conversely, local hormonal treatments have proven efficacy in reversing vulvovaginal atrophy associated with estrogen depletion, although their use in BCSs is controversial due to the risk of increasing estrogen levels.^4,8,9^

In this regard, low-dose local estrogen treatments are considered to have a lower risk profile compared to standard doses and have shown efficacy in several studies in BCSs.^10,11^ Among the variety of local treatments, ultra-low dose of 0.005% estriol vaginal gel has shown a favorable pharmacokinetic and safety profile (ie, a negligible increase of serum estriol levels after three weeks of daily use) and efficacy in reversing vulvovaginal symptoms in healthy postmenopausal women after 12 weeks of treatment.^12,13^ Estriol is a final metabolite unable to convert back to other estrogenic precursors, and has low binding affinity to the estrogen receptor. Furthermore, estriol displays preferential affinity to β (urogenital) rather than α (breast) estrogen receptors. Compared with estradiol, estriol is a less potent hormone with lower estrogenic potential^14^; yet, clinical studies indicate a similar effectiveness of both agents in controlling GSM symptoms.^4,8,15,16^

Even though low-dose estrogen-based vaginal preparations are widely used, scientific evidence regarding their safety and efficacy in women with breast cancer receiving NSAI treatment is still scarce, and the need for placebo-controlled randomized trials in this setting has been highlighted.^14,17,18^ Given that NSAIs are a mainstay of endocrine adjuvant treatment for breast cancer survivors, further clinical research is needed to establish guidelines to treat its associated side effects.^4,8,9,11^ In this randomized, placebo-controlled clinical trial, we investigated the efficacy and safety of an ultra-low dose of 0.005% estriol vaginal gel in postmenopausal women treated with NSAIs under the hypothesis that its application is effective in the management of vulvovaginal symptoms without a significant impact on serum gonadotropins and plasma estrogens.

## MATERIAL AND METHODS

### Study design and population

This was a phase II, randomized, double-blind, placebo-controlled, international, multicenter study for assessing the safety and efficacy of 0.005% estriol vaginal gel in the treatment of symptoms of vulvovaginal atrophy. The study included postmenopausal women with hormone receptor-positive (and any HER2 status) early breast cancer (stage I-IIIA) who had been treated with NSAI (ie, either anastrozole or letrozole) for at least six months. Participants were recruited from five Spanish sites and one Swedish site during a routine follow-up visit between April 2015 and October 2016. Postmenopausal status was defined as 12 months of spontaneous amenorrhea, or 6 months of spontaneous amenorrhea with serum FSH levels greater than 40 mIU/mL, or 6 weeks postsurgical bilateral oophorectomy, with or without hysterectomy. All patients had moderate-to-severe vaginal dryness according to the FDA guidelines for drug development in postmenopausal women (Center for Drug Evaluation and Research, CDER January 2003). A moderate symptom was considered if the symptom was present, bothersome and annoying, and a severe symptom was considered if the symptom was present, bothersome and annoying, and interfered with the normal activity. Other inclusion criteria were a score 0 or 1 in the Eastern Cooperative Oncology Group performance status (ECOG), and an adequate bone marrow and organ function. Patients treated with another antitumoral therapy (excluding pamidronate or alendronate) and those who received vulvovaginal treatment in the 15 days before the study start, and/or had used hormones, natural (phytoestrogens) or herbal products to treat menopausal symptoms in the 3 months before the study start were excluded from the study. Other important exclusion criteria included the presence of postmenopausal uterine bleeding, vaginal bleeding of unknown etiology, and endometrial thickness greater than or equal to 4 mm; and history of other malignancy within 5 years of study entry (aside from nonmelanoma skin cancer or carcinoma-in-situ of the uterine cervix adequately treated), thromboembolic disease or coagulopathies, severe cardiovascular or respiratory disease in the previous 6 months, renal impairment, Hepatitis B and/or C (with the exception of patients with normal hepatic function), and HIV infection. Patients who had received any investigational treatment for any condition or had participated in any clinical trial within 4 weeks of inclusion date were excluded from the study. All patients signed the written informed consent before starting the treatment, and the study protocol was approved by the Regulatory Authorities and applicable Ethics Committees of the participating countries.

### Intervention

The study was conducted in two phases: the safety phase and the study phase. During the safety phase, a sentinel group of 10 women was treated daily for three weeks with active treatment (0.005% estriol vaginal gel, study drug) or placebo (moisturizing gel) at a 4:1 ratio. The influence of treatment on either the levels of systemic estrogens or gonadotropins was assessed by an Independent Data Monitoring Committee. Once the lack of influence on hormone levels was verified, a minimum of 60 women (study group) were randomized to receive either active treatment or placebo at a 4:1 ratio. The study sponsor was responsible for the randomization list, which was performed in blocks of 5. The study drug and placebo had identical characteristics (appearance, smell, and texture), and the investigators and patients were blinded to randomization codes.

Both treatments were administered using an intravaginal applicator at the dose of 1 g of gel per application (containing 50 μg of estriol for the active treatment) for 12 weeks: once daily during the first three weeks, and twice weekly during weeks 4 to 12. During the baseline visit, patients were provided with the investigational products, administered the first dose in the presence of the investigator, and were instructed to administer the gel at bedtime. A screening visit was scheduled within the two weeks prior to the baseline visit. Follow-up visits were performed at weeks 1, 3, 8, and 12 of treatment, and 30 ± 5 days after the last dose (posttreatment visit; only physical examination, general laboratory analysis, and adverse events recording). Treatment compliance was assessed by counting and recording the number of unused applicators.

#### Efficacy and safety assessments

Efficacy assessments were performed at baseline, and weeks 3 and 12, and included a gynecological examination and the administration of the Female Sexual Function Index (FSFI) questionnaire. Vaginal symptoms (ie vaginal dryness, dyspareunia, and pruritus) and signs (ie mucosa with thinning or flattening of folds, and fragility and dryness of the mucosa) were rated by the patient (symptoms) and the investigator (signs) on a 4-point scale, where 0 indicated the absence of the sign/symptom and 3 indicated the symptom was considered very irritating and severe; scores 1 and 2 indicated mild and moderate signs/symptoms, respectively The vaginal pH was measured on the vaginal secretion using a reactive strip (pH 2.0-9.0). Vaginal maturation was assessed from two cytology samples (drawn from the bottom of the right and left vaginal sac, respectively), and were analyzed at a central laboratory (Centro Anatomopatológico, S.L., Madrid, Spain). For the cytological evaluation, the average percentages of parabasal, intermediate, and superficial cells were calculated twice on 100 consecutive cells. The maturation index was determined using the formula 0.2*x* (% parabasal) + 0.6*x* (% intermediate) + 1.0*x* (% superficial) (range 20-100).^19^ The FSFI is a 19-item questionnaire, composed of three multi-item functional subscales covering six domains (ie, desire, arousal, lubrication, orgasm, satisfaction, and pain), developed specifically to assess sexual functioning in the research setting.^20^ An algorithm was used to determine domain scores and a composite full-scale score.

Safety was assessed based on the occurrence of adverse events, endometrial changes from baseline to week 12 as evidenced by ultrasound, and the changes in the serum levels of gonadotropins (FSH and luteinizing hormone [LH]) and plasma levels of estrogens (estriol, estradiol, and estrone) throughout treatment. Estrogen levels were determined at a central laboratory (Pharm-Analyt, Austria) at baseline and weeks 1, 3, 8, and 12. Due to the extremely low levels of estrogens expected, estriol, estradiol, and estrone were analyzed using a validated ultrasensitive liquid chromatography tandem mass spectrometry (LC-MS/MS) method. Values below limit of quantification (1, 3 and 5 pg/ml) were considered 0.5, 1.5, and 2.5 pg/mL for estriol, estradiol, and estrone, respectively. FSH and LH were analyzed by chemiluminescent immunoassay at Laboratorios Echevarne, Spain at the same time points and, additionally, at the screening visit to assess their physiological variability. Determinations for a given participant were performed in the same batch. Other safety assessments included laboratory parameters (hematology, blood chemistry, and urine tests) and a physical and gynecological examination (breast and pelvic examination), performed at baseline and at weeks 3 and 12. All adverse events were recorded and graded according to the Medical Dictionary for Regulatory Activities. The causal relationship between the investigational product and the adverse event was assessed by the investigator using the Karch and Lasagna algorithm.^21^

### Statistics

Analyses regarding the change in gonadotropins were performed in the intent-to-treat (ITT) population, which included all 61 randomized participants; missing data were imputed using the last observation carried forward method. Data missing in the efficacy analysis were not imputed. Categorical variables were presented as frequency and percentage, whereas quantitative variables were presented as the mean and standard deviation (SD) or the median and interquartile range (IQR). Results regarding the progression of signs and symptoms severity throughout the follow-up period were presented as both mean (SD) and median (IQR) to provide a comprehensive view of que quantitative change, which in some cases occurred in a narrow score range. Because of the high variability of physiological levels of gonadotropins, the change in these hormones was assessed by comparing the physiological variability (ie, difference between screening and baseline visits) and the in-treatment variability (ie, difference from baseline to 12-week treatment). The between-group differences in score change were compared using the nonparametric Mann-Whitney-*U* test, whereas differences between time points within each treatment group were compared using the Wilcoxon signed-rank test (results are presented as mean and SD) All analyses were performed using the statistical software SAS Enterprise Guide 5.1.

## RESULTS

### Characteristics of Study Patients

**FIG. 1:**
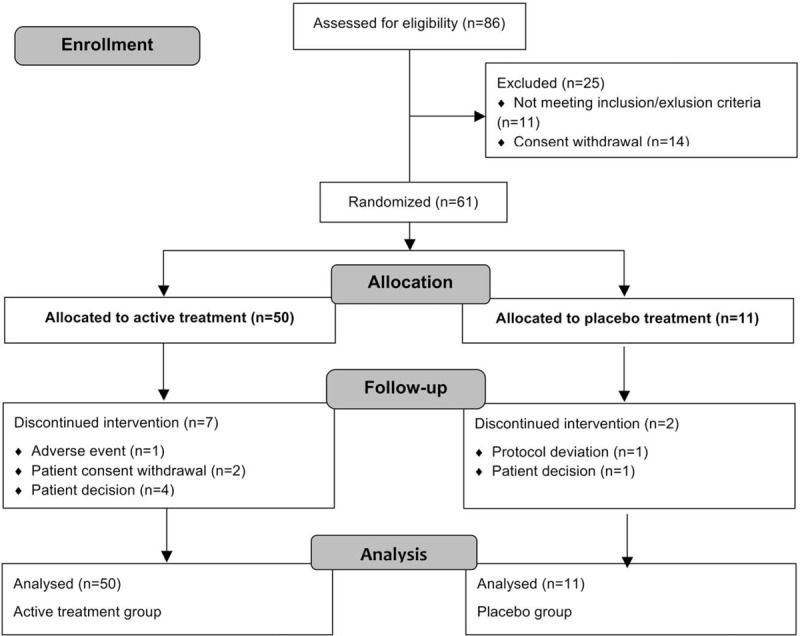
Flow diagram of the patients included in the study.

Of 86 patients considered for eligibility, 61 were randomized to receive either 0.005% estriol vaginal gel (*n* = 50) or placebo moisturizing gel (*n* = 11) (Fig. 1). Nine patients, seven in the active group and two in the placebo group, discontinued the intervention but were nevertheless included in the analysis of the ITT population. The ITT population included postmenopausal women with mean (SD) age of 58.9 (7.6) and 61.4 (4.7) years (active and placebo groups, respectively). Twenty-one (34.4%) and 40 (65.6%) women complained of moderate- and severe-vaginal dryness, respectively; 12 (19.7%) and 33 (54%) from moderate and severe dyspareunia, respectively; and 36 (59%) from mild-to-severe pruritus. Table 1 summarizes the baseline demographic and clinical characteristics of the study participants. At baseline, patients in the two groups showed no significant differences regarding vaginal pH and maturation, the scores of the symptoms (ie, dyspareunia, pruritus, and vaginal dryness) and signs (ie, dryness, fragility of the mucosa, and vaginal mucosa with flattening of folds or thinning) of vaginal atrophy (Table 2). Likewise, the scores of the FSFI questionnaire (total and for each domain) were similar between the two study groups.

**TABLE 1:** Baseline demographic, clinical and previous treatment characteristics of the study patients, *n* (%)

	Active (*n* = 50)	Placebo (*n* = 11)	Total (*n* = 61)
Postmenopausal status			
≥12 months of spontaneous amenorrhea	44 (88.0)	11 (100.0)	55 (90.2)
At least 6 weeks postsurgical bilateral oophorectomy^*a*^	5 (10.0)	0 (0.0)	5 (8.2)
6 months of spontaneous amenorrhea with serum FSH increased levels > 40 mIU/mL	1 (2.0)	0 (0.0)	1 (1.6)
ECOG performance status			
0	44 (88.0)	11 (100.0)	55 (90.2)
1	5 (10.0)	0 (0.0)	5 (8.2)
Not available	1 (2.0)	0 (0.0)	1 (1.6)
Breast cancer stage			
I	18 (36.0)	4 (36.4)	22 (36.1)
IIA	23 (46.0)	3 (27.3)	26 (42.6)
IIB	7 (14.0)	2 (18.2)	9 (14.8)
IIIA	2 (4.0)	2 (18.2)	4 (6.6)
Estrogen receptor			
Positive	50 (100.0)	11 (100.0)	61 (100.0)
Progesterone receptor			
Negative	11 (22.0)	2 (18.2)	13 (21.3)
Positive	38 (76.0)	9 (81.8)	47 (77.0)
Not available	1 (2.0)	0 (0.0)	1 (1.6)
HER2			
Negative	37 (74.0)	8 (72.7)	45 (73.8)
Positive	13 (26.0)	3 (27.3)	16 (26.2)
Previous adjuvant hormonal treatments			
Aromatase inhibitors	41 (82.0)	10 (90.9)	51 (83.6)
Aromatase inhibitors+tamoxifen	8 (16.0)	1 (9.1)	9 (14.8)
Aromatase inhibitors+tamoxifen+LHRH agonist	1 (2.0)	0 (0.0)	1 (1.6)

ECOG, Eastern Cooperative Oncology Group; HER2, human epidermal growth factor receptor 2; LHRH, luteinizing hormone-releasing hormone.

^*a*^Including patients with and without hysterectomy.

**TABLE 2:** Baseline scores of symptoms and signs of vaginal atrophy and FSFI scores, according to treatment allocation, median (percentile 25.75)

	Active	Placebo	*P* value^*a*^
Maturation value	24.6 (21.3,30.8)	23.4 (21.0,24.6)	0.180
Vaginal pH	6.5 (5.5,7.5)	7.0 (5.5,8.0)	0.550
Symptoms of vaginal atrophy			
Dyspareunia	3.0 (2.0,3.0)	3.0 (2.0,3.0)	0.100
Pruritus	1.0 (0.0,2.0)	0.0 (0.0,2.0)	0.540
Vaginal dryness	3.0 (2.0,3.0)	3.0 (2.0,3.0)	0.590
Total score	6.0 (6.0,7.0)	6.0 (6.0,8.0)	0.680
Signs of vaginal atrophy			
Dryness	2.0 (2.0,3.0)	3.0 (2.0,3.0)	0.140
Fragility of the mucosa	2.0 (1.0,3.0)	2.0 (2.0,3.0)	0.520
Vaginal mucosa with flattening of folds or thinning	2.0 (2.0,3.0)	2.0 (2.0,2.0)	0.130
Total score	7.0 (5.0,8.0)	7.0 (6.0,8.0)	0.710
FSFI			
Desire	1.2 (1.2,2.4)	1.2 (1.2,2.4)	1.000
Arousal	1.5 (0.0,2.7)	1.8 (0.0,2.7)	0.820
Lubrication	1.2 (0.0,2.4)	1.4 (0.0,2.4)	0.980
Orgasm	1.2 (0.0,2.4)	2.6 (0.0,4.8)	0.320
Satisfaction	2.4 (2.0,3.2)	2.4 (2.4,4.0)	0.460
Pain	0.8 (0.0,5.2)	2.4 (0.0,5.2)	0.880
Total Score	14.3 (5.4,18.4)	18.3 (5.1,22.4)	0.470

FSFI, Female Sexual Function Index.

^*a*^Mann-Whitney-*U* test.

### Efficacy on maturation value and vaginal pH

**FIG. 2:**
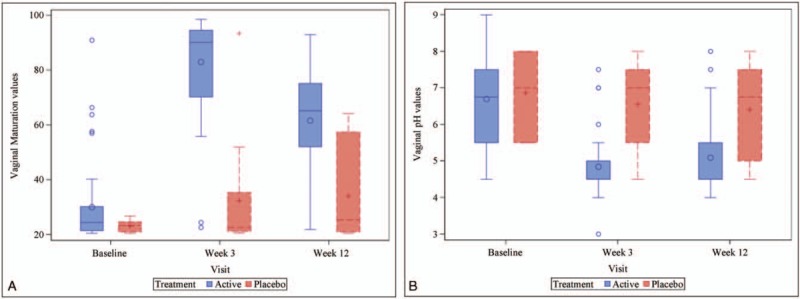
Boxplot of the vaginal maturation (**A**) and vaginal pH (**B**) values determined at each of the indicated visits in the active treatment and placebo groups (ITT population). ITT, intent-to-treat.

The median (IQR) vaginal maturation scoring in the active group increased significantly from 24.6 (21.3, 30.8) at baseline to 90.2 (72.8, 94.6) at week 3 and 65.2 (51.4, 75.0) at week 12 (*P < *0.001 for each comparison with baseline), and remained similar in the placebo group (*P = *0.717 and *P = *0.244, for differences between baseline and weeks 3 and 12, respectively) (Fig. 2). Correspondingly, vaginal maturation values were significantly higher in the active compared to the placebo treatment arm: mean (SD) for active and placebo groups were 83.1 (17.7) and 32.4 (22.4) at week 3, and 61.0 (21.2) and 34.1 (18.1) at week 12, respectively (*P < *0.001, for each time point). A similar trend was observed for vaginal pH: patients in the active group experienced a significant decrease in median vaginal pH from baseline (6.5, IQR 5.5, 7.5) to week 3 (4.5, IQR 4.5, 5.0) and week 12 (4.5, IQR 4.5, 5.5) (*P < *0.001, for each comparison with baseline). Conversely, no significant differences were found between baseline and week 3 and week 12 in the placebo group (*P = *0.38 and *P = *0.184, respectively) (Fig. 2). Likewise, pH was significantly lower in the active compared to the placebo treatment arm, with mean (SD) values of 4.8 (0.8) and 6.5 (1.1) at week 3 (*P < *0.001), and 5.1 (0.9) and 6.4 (1.4) at week 12 (*P = *0.004), respectively.

### Efficacy of symptoms and signs of vulvovaginal atrophy

**FIG. 3:**
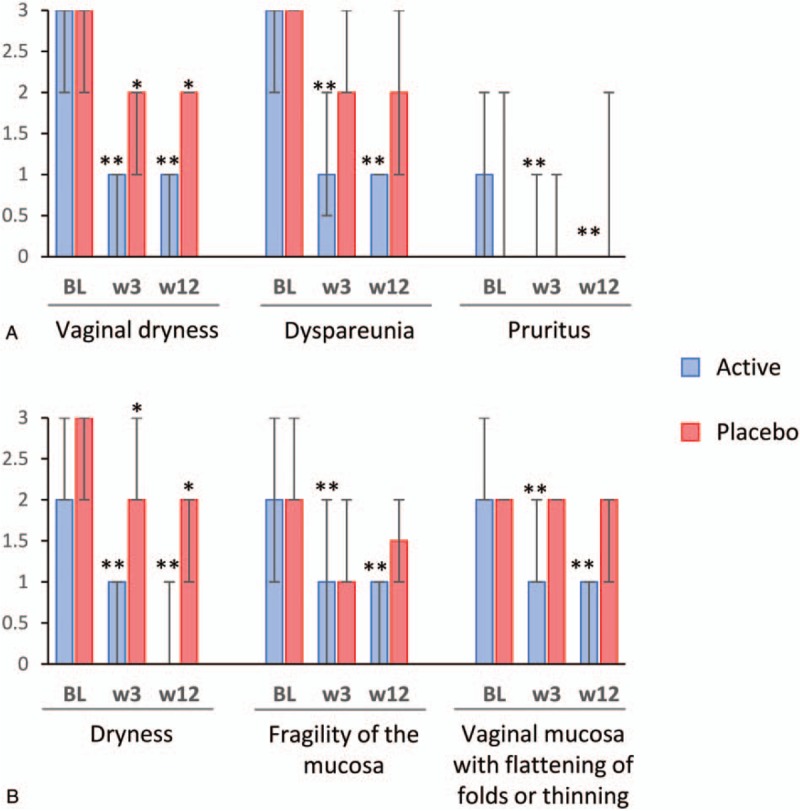
Individual scores of each symptom (**A**) and signs (**B**) of vulvovaginal atrophy throughout treatment. Columns and error bars represent median and IQR, respectively. Symptoms/signs severity are presented on a 0-3 scale: 0 = lack of symptoms/signs, 1 = mild, 2 = moderate, and 3 = severe. BL, baseline; IQR, interquartile range; w, week. ^∗∗^*P < *0.001, ^∗^*P < *0.05 for differences between baseline and the given visit.

**FIG. 4:**
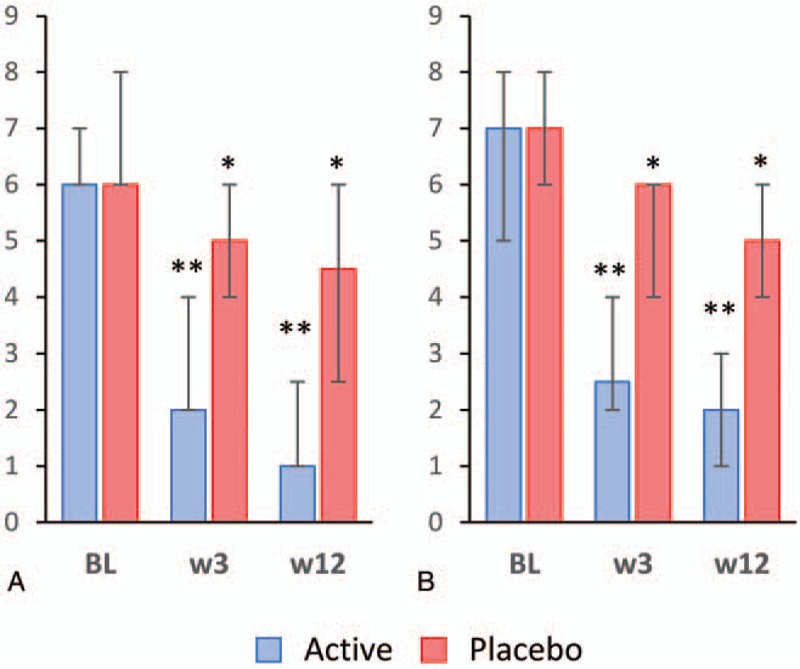
Total scores of symptoms (**A**) and signs (**B**) of vulvovaginal atrophy throughout treatment. Columns and error bars represent median and IQR, respectively. BL, baseline; IQR, interquartile range; w, week. ^∗∗^*P < *0.001, ^∗^*P < *0.05 for differences between baseline and the given visit.

Patients in the active group, but not those in the placebo group, experienced a significant decrease in the scores of dyspareunia, pruritus, fragility of the mucosa, and mucosa with flattening of folds or thinning between baseline, week 3, and week 12. Conversely, the scores for vaginal dryness (symptom) and dryness (sign) significantly decreased in the active and, to a minor extent, in the placebo group after 3 and 12 weeks of treatment (Fig. 3). A similar trend was observed for the total scores of symptoms and signs of vaginal atrophy, which significantly decreased in the active and, to a minor extent, in the placebo group at the follow-up visits (Fig. 4). The extent of the change in the scores of vulvovaginal symptoms and signs in the active and placebo groups throughout the follow-up period is summarized in Table 3. Changes in total symptom score between baseline and the two follow-up visits were significantly greater in the active group compared to the placebo group. Regarding individual symptoms, the assessment of vaginal dryness at week 12 yielded significant differences between groups. On the other hand, all the signs analyzed and the total signs score showed statistically significant differences between active and placebo groups at week 3 and week 12, except for fragility of the mucosa, whose changes were statistically significant only at week 12.

**TABLE 3:** Changes in vaginal maturation value, vaginal pH, and the scores of symptoms and signs of vaginal atrophy

	Baseline-week 3			Baseline-week 12		
	Active (*n* = 44)	Placebo (*n* = 11)	*P* value	Active (*n* = 45)	Placebo (*n* = 10)	*P* value
Maturation value	−53.4 (21.9)	−9.2 (22.4)		−31.8 (23.8)	−10.9 (18.5)	
	−62.0 (−70.4,−37.8)	−0.6 (−11.6,2.0)	**<0.0001**	−37.2 (−50.0,−5.4)	−1.0 (−36.4,1.4)	**<0.01**
Vaginal pH	1.8 (1.3)	0.3 (1.3)		1.6 (1.4)	0.5 (1.4)	
	2.0 (0.5,2.5)	0.5 (−0.5,1.0)	**<0.001**	1.3 (0.5,2.5)	0.5 (0.0,1.5)	0.058
Symptoms						
Vaginal dryness	1.3 (0.9)	0.8 (0.9)		1.8 (0.9)	0.9 (1.0)	
	1.0 (1.0,2.0)	1.0 (0.0,1.0)	0.14	2.0 (1.0,2.0)	1.0 (1.0,1.0)	**<0.01**
Dyspareunia	1.3 (0.9)	0.8 (0.7)		1.6 (1.0)	1.1 (1.1)	
	1.0 (1.0,2.0)	1.0 (0.0,1.0)	0.14	2.0 (1.0,2.5)	1.0 (0.0,2.0)	0.25
Pruritus	0.5 (1.0)	0.2 (1.3)		0.7 (1.3)	0.3 (1.2)	
	0.0 (0.0,1.0)	0.0 (−1.0,2.0)	0.29	0.0 (0.0,2.0)	0.0 (0.0,1.0)	0.34
Total score symptoms	3.5 (2.0)	1.8 (1.8)		4.6 (2.1)	2.6 (2.4)	
	3.0 (2.0,4.5)	1.0 (0.5,3.0)	**0.03**	4.5 (3.0,7.0)	2.0 (1.5,4.0)	**0.04**
Signs						
Dryness of the mucosa	1.7 (0.8)	0.7 (0.6)		1.7 (0.9)	0.8 (0.8)	
	2.0 (1.0,2.0)	1.0 (0.0,1.0)	**<0.001**	2.0 (1.0,2.0)	1.0 (0.0,1.0)	**<0.01**
Fragility of the mucosa	1.0 (0.9)	0.5 (0.9)		1.4 (0.8)	0.5 (0.5)	
	1.0 (0.0,2.0)	0.0 (0.0,1.0)	0.13	1.5 (1.0,2.0)	0.5 (0.0,1.0)	**<0.01**
Mucosa with thinning or flattening of folds	1.1 (0.8)	0.2 (0.4)		1.5 (0.9)	0.3 (0.5)	
	1.0 (1.0,2.0)	0.0 (0.0,0.0)	**<0.001**	1.0 (1.0,2.0)	0.0 (0.0,1.0)	**<0.001**
Total score signs	3.8 (1.9)	1.5 (1.4)		4.6 (2.1)	1.6 (1.5)	
	4.0 (2.0,5.0)	1.0 (0.0,2.0)	**<0.001**	5.0 (4.0,6.0)	1.0 (1.0,3.0)	**<0.001**

All values in bold are considered significant according to the threshold established for this analysis. Results are described as mean (standard deviation) (upper row) and median (percentile 25.75) (lower row). Between-group differences were analyzed using the Mann-Whitney-*U* test.

### Impact on sexual function

Although patients in the placebo group did not show significant differences after treatment, those in the active group experienced a progressive and significant increase in the FSFI total score from baseline, a trend that was maintained in the individual analysis of the desire, arousal, lubrication, orgasm, and satisfaction domains, but not in the pain domain (Table 4). Despite the significant improvement in most domains observed only in the active group, the pairwise comparison at each visit did not reveal significant differences between active and placebo groups regarding the total FSFI score and the scores of each domain.

**TABLE 4:** FSFI domain and total scores throughout treatment, median (percentile 25.75)

	Baseline (*N* = 57)	Week 3 (*N* = 51)	Week 12 (*N* = 55)	*P* value^*a*^ BL-w3	*P* value^*a*^ BL-w12
Domains				
Desire				
Active	1.2 (1.2,2.4)	2.4 (1.2,3.0)	2.4 (1.2,3.6)	**<0.001**	**<0.001**
Placebo	1.2 (1.2,2.4)	2.1 (1.2,2.4)	2.4 (1.2,3.0)	0.563	0.484
Arousal				
Active	1.5 (0.0,2.7)	2.1 (0.6,3.6)	2.7 (0.9,4.8)	**<0.001**	**<0.001**
Placebo	1.8 (0.0,2.7)	1.7 (0.0,3.0)	2.7 (0.3,3.6)	0.688	1.000
Lubrication				
Active	1.2 (0.0,2.4)	3.0 (0.0,4.8)	3.3 (0.0,5.1)	**<0.001**	**<0.001**
Placebo	1.4 (0.0,2.4)	2.1 (0.0,2.4)	2.3 (0.3,5.1)	0.594	0.234
Orgasm				
Active	1.2 (0.0,2.4)	2.4 (0.0,4.4)	3.2 (0.0,4.4)	**0.008**	**<0.001**
Placebo	2.6 (0.0,4.8)	2.0 (0.0,2.8)	3.8 (0.0,5.2)	0.594	0.680
Satisfaction				
Active	2.4 (2.0,3.2)	3.8 (2.2,5.0)	4.8 (3.4,5.4)	**<0.001**	**<0.001**
Placebo	2.4 (2.4,4.0)	3.8 (2.4,4.8)	4.2 (2.0,5.2)	0.469	0.391
Pain				
Active	0.8 (0.0,5.2)	1.4 (0.0,3.4)	1.2 (0.0,3.6)	0.117	0.157
Placebo	2.4 (0.0,5.2)	2.4 (0.0,4.8)	1.6 (0.0,4.4)	1	0.563
Total score				
Active	14.3 (5.4,18.4)	18.0 (6.0, 24.2)	21.7 (17.7, 26.0)	**0.007**	**<0.001**
Placebo	18.3 (5.1,22.4)	18.0 (10.4,20.2)	20.1 (5.0,23.7)	0.875	0.438

All values in bold are considered significant according to the threshold established for this analysis. BL, baseline; FSFI, Female Sexual Function Index.

^*a*^Signed rank test (Wilcoxon).

### Safety

Patients treated with estriol gel did not experience significant changes in FSH levels (median difference between physiological and in-treatment variability after 12 weeks of treatment was −2.8 [IQR −13.1; 7.4]; *P = *0.104) and LH levels (−0.8 [IQR −5.3; 2.9]; *P = *0.116; *N* = 61). In the active group, median estriol levels slightly increased at week 1 and progressively normalized over the treatment period. The levels of the key hormones at each study visit are summarized in Supplementary Table 1 (Supplementary file 1). The median and IQR of both estrone and estradiol were persistently below the limit of quantification at all study visits for both groups. During the 16 weeks of follow-up (including the posttreatment visit), no deaths or serious adverse events (SAEs) related to the treatment were reported. An SAE unrelated to the study treatment consisting of non-Hodgkin's lymphoma appeared before the administration of the study treatment and resulted in study discontinuation. Eight nonserious adverse events were reported once each in six patients in the active group: breast tenderness, vulvovaginal inflammation, vulvovaginal pruritus, burning sensation, diarrhea, vomiting, mucosal dryness, and pyrexia.

## DISCUSSION

In this phase II, randomized, double-blind, placebo-controlled clinical trial, we provide scientific evidence regarding the efficacy and safety of 0.005% estriol vaginal gel for the treatment of genital symptoms in postmenopausal women receiving NSAI adjuvant therapy for hormone receptor-positive early breast cancer. After 12 weeks of treatment, the local application of the ultra-low dose of estriol gel reversed most of the vulvovaginal symptoms and signs associated with deep estrogen deprivation in BSCs receiving adjuvant treatment, including the most common and bothersome symptoms (ie vaginal dryness and dyspareunia), resulting in a positive impact on sexual function.

Vulvovaginal symptoms of menopause are a highly prevalent side effect of adjuvant hormonal treatments in BCSs, particularly in women receiving NSAIs, who experience vaginal dryness more frequently compared to women receiving tamoxifen.^6^ In spite of the widespread use of local estrogens for reversing this and other symptoms and signs of vulvovaginal atrophy in healthy postmenopausal women, clinical evidence supporting the safety and efficacy of local application of estrogens in BCSs is scarce.^8,10,22^ Our results regarding the efficacy of the product in reducing the signs/symptoms burden were similar to those of previous trials investigating other estrogen formulations containing either estradiol or estriol at higher doses such as estradiol ring and tablets (7.5 and 12.5 μg, respectively), estriol ovules (0.5 mg) and cream (0.25 mg), and estriol tablets (0.03 mg) containing *Lactobacillus acidophilus*.^3,23,24^ In spite of the lesser estrogenic action of estriol (compared to estradiol) and the 10-fold lower dose (compared to other estriol products widely indicated for this use), the ultra-low dose of estriol used in this study (50 μg per application dose) showed efficacy in reversing the changes in the vaginal epithelium associated with the deep estrogen deprivation in postmenopausal BCSs receiving NSAIs, providing clinical evidence that this population could benefit from local estrogen treatment.

Interestingly, although the intensity of most symptoms and signs of vaginal atrophy remained unchanged in the placebo treatment arm, the scores of vaginal dryness (sign and symptom) experienced a significant—albeit modest— reduction after 3 and 12 weeks of placebo treatment. Given that the 0.005% estriol gel is formulated as a highly hydrating gel with a true effect beyond the pure placebo effect, treatment with the gel alone (without hormones) was expected to have an effect on some of the symptoms and signs (ie temporal relief of vaginal dryness), similar to the lubricants and moisturizers indicated as first-line treatment for vaginal atrophy.^2,4,8^ Nevertheless, patients in the active arm experienced a greater improvement in vaginal dryness throughout the follow-up, likely due to the stimulation of maturation of the vaginal epithelium by the active hormone and its healing effect. Regarding sexual function, only the 0.005% estriol gel improved most domains and the total score of the FSFI questionnaire, whereas the placebo hydrating gel failed to improve sexual function. Given the predominant impact of NSAIs treatment on sexual function and consequently, well-being and quality of life, these findings suggest that the ultra-low 0.005% estriol vaginal gel may improve patients’ quality of life in addition to alleviating GSM symptoms.^25^

Although studies with local estrogens in BCSs receiving NSAIs are scarce, the efficacy of local estrogen treatment, such as low-dose estriol, in alleviating symptoms and signs of vulvovaginal atrophy in healthy postmenopausal women is well documented.^26^ In this regard, a previous pivotal phase III placebo-controlled study including 167 women reported efficacy of the 0.005% estriol ultra-low dose vaginal gel.^12^ Hence, irrespective of the cause of estrogen deprivation (ie, natural vs NSAIs-associated), the 0.005% estriol gel effectively reversed the changes in the vaginal epithelium associated with estrogen deprivation.

In spite of the randomized placebo-controlled nature of this trial, our analysis was constrained to a 12-week follow-up, thus precluding long-term assessment of the effects of estriol. Some authors have reported an increased absorption in atrophic compared to estrogenized vaginal tissues and a differential expression of estrogen receptors in the vaginal walls of pre- and post-menopausal women.^27,28^ Although these observations likely rule out increased absorption of estriol by the well estrogenized epithelium and, consequently, safety concerns after prolonged treatment, long-term studies may be needed to assess the sustained safety and effectivity throughout the prescribed adjuvant treatment. It is also worth mentioning that the scarcity of previous data regarding the change of FSH in patients treated with estriol vaginal gels precluded a formal estimation of sample size; however, the results of a previous analysis of FSH change in these patients suggested that the size of our sample should be large enough to identify significant changes in FSH levels.^24^ Given that our results indicate that the 0.005% estriol gel preparation is effective and safe, further studies using a larger sample size (with a balanced randomization) to assess long-term effects are warranted.

## CONCLUSIONS

Our results provide clinical evidence on the efficacy of 0.005% estriol vaginal gel in postmenopausal breast cancer survivors treated with NSAI. Despite the ultra-low dose of estriol, the application of the gel is safe and improves vaginal symptoms and signs, including vaginal pH and maturation, associated with the depletion of estrogens that occurs in these patients, resulting in improved sexual function. The negligible impact of the product on the levels of estrogens, FSH, and LH supports the safe use of this ultra-low dose estriol vaginal gel as a treatment option for vulvovaginal atrophy in BSCs receiving NSAIs.

## Supplementary Material

Supplemental Digital Content

### Supplementary Material

Supplemental Digital Content
