# Supplementary material for: Efficacy and safety of ultra-low dose 0.005% estriol vaginal gel for the treatment of vulvovaginal atrophy in postmenopausal women with early breast cancer treated with nonsteroidal aromatase inhibitors: a phase II, randomized, double-blind, placebo-controlled trial
Source: Menopause. 2020 Feb 10;27(5):526–34. doi: 10.1097/GME.0000000000001497 (PMC7188038; doi:10.1097/GME.0000000000001497)
Supplement: Supplemental Digital Content [file menop-27-526-s002.docx]

**Efficacy and Safety of Ultra-Low-Dose 0.005% Estriol Vaginal Gel for the Treatment of Vulvovaginal Atrophy in Postmenopausal Women with Early Breast Cancer Treated with Non-Steroidal Aromatase Inhibitors: A Phase II, Randomized, Double-Blind, Placebo-Controlled Trial**

Supplementary file 1

| **Table S1.** Hormone levels at each study visit. | | | |
| --- | --- | --- | --- |
|  | | **Active** | **Placebo** |
|  |  | **( N = 50 )** | **( N = 11 )** |
| **FSH** | | | |
| **Screening: FSH Values (LOCF)** | | | |
| Median |  | 55.3 | 52 |
| Q1 ; Q3 |  | 45.5 ; 68.5 | 45.7 ; 83.1 |
| **Baseline: FSH Values (LOCF)** | | | |
| Median |  | 54.6 | 47.6 |
| Q1 ; Q3 |  | 42.2 ; 67.8 | 35.6 ; 89.6 |
| **Mean (Screening, Baseline): FSH Values (LOCF)** | | | |
| Median |  | 55.8 | 49.3 |
| Q1 ; Q3 |  | 43.4 ; 66.5 | 43.8 ; 85.5 |
| **Week 1: FSH Values (LOCF)** | | | |
| Median |  | 47.6 | 53.8 |
| Q1 ; Q3 |  | 37.5 ; 59.5 | 51.4 ; 83.5 |
| **Week 3: FSH Values (LOCF)** | | | |
| Median |  | 48.7 | 65.1 |
| Q1 ; Q3 |  | 41.2 ; 59.0 | 44.4 ; 75.8 |
| **Week 8: FSH Values (LOCF)** | | | |
| Median |  | 51.4 | 55.7 |
| Q1 ; Q3 |  | 37.5 ; 62.5 | 46.8 ; 83.3 |
| **Week 12: FSH Values (LOCF)** | | | |
| Median |  | 51 | 62.1 |
| Q1 ; Q3 |  | 38.6 ; 63.5 | 46.8 ; 90.1 |
| **LH** | | | |
| **Screening: LH Values (LOCF)** | | | |
| Median |  | 21 | 24.7 |
| Q1 ; Q3 |  | 13.9 ; 29.5 | 17.6 ; 35.7 |
| **Baseline: LH Values (LOCF)** | | | |
| Median |  | 19.8 | 25.3 |
| Q1 ; Q3 |  | 14.8 ; 30.9 | 15.4 ; 39.3 |
| **Mean (Screening, Baseline): LH Values (LOCF)** | | | |
| Median |  | 18.7 | 25 |
| Q1 ; Q3 |  | 14.0 ; 30.7 | 16.5 ; 38.4 |
| **Week 1: LH Values (LOCF)** | | | |
| Median |  | 18.2 | 30.1 |
| Q1 ; Q3 |  | 13.7 ; 25.1 | 18.9 ; 36.5 |
| **Week 3: LH Values (LOCF)** | | | |
| Median |  | 19.6 | 28.7 |
| Q1 ; Q3 |  | 15.1 ; 27.0 | 20.5 ; 36.9 |
| **Week 8: LH Values (LOCF)** | | | |
| Median |  | 17.7 | 27.8 |
| Q1 ; Q3 |  | 13.9 ; 26.2 | 21.4 ; 33.5 |
| **Week 12: LH Values (LOCF)** | | | |
| Median |  | 19.3 | 28.9 |
| Q1 ; Q3 |  | 14.4 ; 27.6 | 20.0 ; 38.0 |
| **Estriol ^a^** | | | |
| **Baseline: Estriol Values (LOCF)** | | | |
| Median |  | 0.5 | 0.5 |
| Q1 ; Q3 |  | 0.5 ; 1.0 | 0.5 ; 0.5 |
| **Week 1: Estriol Values (LOCF)** | | | |
| Median |  | 3.9 | 0.5 |
| Q1 ; Q3 |  | 0.5 ; 12.1 | 0.5 ; 0.5 |
| **Week 3: Estriol Values (LOCF)** | | | |
| Median |  | 1.9 | 0.5 |
| Q1 ; Q3 |  | 0.5 ; 6.8 | 0.5 ; 0.5 |
| **Week 8: Estriol Values (LOCF)** | | | |
| Median |  | 0.5 | 0.5 |
| Q1 ; Q3 |  | 0.5 ; 6.0 | 0.5 ; 0.5 |
| **Week 12: Estriol Values (LOCF)** | | | |
| Median |  | 0.5 | 0.5 |
| Q1 ; Q3 |  | 0.5 ; 7.3 | 0.5 ; 0.5 |

**LOCF:** Last observation carried forward

^a^ Determinations below the limit of quantification were assigned to 0.5 pg/mL
